# AnnQ: reference-based quantification of cellular abnormality at single-cell resolution

**DOI:** 10.1093/bib/bbag278

**Published:** 2026-05-31

**Authors:** Davin Lee, Gaeun Byeon, Seojin Chung, Dongmin Shin, Jongseo Park, Ingyeon Koh, Joon-Yong An

**Affiliations:** Department of Integrated Biomedical and Life Science, Korea University, 145 Anam-ro, Seongbuk-gu, Seoul 02841, Republic of Korea; Interdisciplinary Major Program in Targeted Degradation-based Innovative Therapeutics, Korea University, 145 Anam-ro, Seongbuk-gu, Seoul 02841, Republic of Korea; National Research Laboratory for Convergence Degradation Biology, Korea University, 145 Anam-ro, Seongbuk-gu, Seoul 02841, Republic of Korea; Department of Integrated Biomedical and Life Science, Korea University, 145 Anam-ro, Seongbuk-gu, Seoul 02841, Republic of Korea; National Research Laboratory for Convergence Degradation Biology, Korea University, 145 Anam-ro, Seongbuk-gu, Seoul 02841, Republic of Korea; L-HOPE Program for Community-Based Total Learning Health Systems, Korea University, Republic of Korea, Republic of Korea; School of Biosystem and Biomedical Science, College of Health Science, Korea University, Republic of Korea, Republic of Korea; Department of Integrated Biomedical and Life Science, Korea University, 145 Anam-ro, Seongbuk-gu, Seoul 02841, Republic of Korea; National Research Laboratory for Convergence Degradation Biology, Korea University, 145 Anam-ro, Seongbuk-gu, Seoul 02841, Republic of Korea; L-HOPE Program for Community-Based Total Learning Health Systems, Korea University, Republic of Korea, Republic of Korea; School of Health and Environmental Science, College of Health Science, Korea University, 145 Anam-ro, Seongbuk-gu, Seoul 02841, Republic of Korea; Department of Integrated Biomedical and Life Science, Korea University, 145 Anam-ro, Seongbuk-gu, Seoul 02841, Republic of Korea; National Research Laboratory for Convergence Degradation Biology, Korea University, 145 Anam-ro, Seongbuk-gu, Seoul 02841, Republic of Korea; L-HOPE Program for Community-Based Total Learning Health Systems, Korea University, Republic of Korea, Republic of Korea; Genetics and Genome Biology Program, The Hospital for Sick Children; Toronto, ON M5G 0A4, Canada; The Centre for Applied Genomics, The Hospital for Sick Children; Toronto, ON M5G 0A4, Canada; Department of Integrated Biomedical and Life Science, Korea University, 145 Anam-ro, Seongbuk-gu, Seoul 02841, Republic of Korea; Interdisciplinary Major Program in Targeted Degradation-based Innovative Therapeutics, Korea University, 145 Anam-ro, Seongbuk-gu, Seoul 02841, Republic of Korea; National Research Laboratory for Convergence Degradation Biology, Korea University, 145 Anam-ro, Seongbuk-gu, Seoul 02841, Republic of Korea; L-HOPE Program for Community-Based Total Learning Health Systems, Korea University, Republic of Korea, Republic of Korea; School of Biosystem and Biomedical Science, College of Health Science, Korea University, Republic of Korea, Republic of Korea

**Keywords:** single-cell RNA sequencing, cell type annotation, annotation uncertainty, reference-based analysis, perturbation analysis, out-of-reference score

## Abstract

Reference-based annotation tools have become standard for cell type assignment in single-cell RNA sequencing, leveraging large-scale atlases to transfer labels to new datasets. However, a substantial fraction of cells often receive uncertain or ambiguous annotations—low confidence scores, competing label probabilities, or high entropy across cell types. These cells are typically treated as technical artifacts and filtered out, yet in perturbation experiments they may represent the biologically interesting deviations that investigators seek to identify. We present AnnQ (Annotation Quantification of cellular identity uncertainty), a Python framework that repurposes annotation uncertainty as a quantitative measure of cellular abnormality. AnnQ extracts uncertainty-aware features from probabilistic cell type assignments—including confidence, confidence gap, admixture ratio, and entropy—and computes an out-of-reference (OOR) score measuring each cell’s deviation from a reference population in multivariate uncertainty space. Applying AnnQ to genetic perturbation and drug resistance datasets, we show that OOR scores detect aberrant cellular states that are not resolved by conventional clustering or differential abundance analyses. AnnQ provides a complementary approach for characterizing transitional and abnormal cell states at single-cell resolution. AnnQ is implemented in Python, and its source code and documentation are available on https://github.com/joonan-lab/AnnQ.git.

Key Points

- Reference-based annotation tools generate probability distributions over cell types, but current workflows discard this distributional information after extracting discrete labels, missing biologically meaningful signals encoded in annotation uncertainty.
- AnnQ transforms annotation uncertainty into a multivariate feature space and computes an out-of-reference (OOR) score measuring each cell’s deviation from a normative reference population, providing a continuous quantification of cellular abnormality.
- In genetic perturbation models, AnnQ detects coordinated identity destabilization that does not form separable clusters in standard analyses, revealing graded states of cellular aberrancy invisible to conventional approaches.
- In longitudinal cancer datasets, AnnQ tracks drug-tolerant persister-to-metastatic precursor transitions through annotation dynamics alone, demonstrating that uncertainty features encode disease-state information beyond what discrete labels capture.
- Cross-atlas validation across five independent cancer cohorts confirms reproducibility of known instability patterns and uncovers previously unappreciated multi-axis deviation structures, establishing annotation uncertainty as a systematic discovery tool.

## Introduction

Over the past few years, the single-cell research community has witnessed the emergence of mega-scale single-cell RNA-seq atlases, driven by extensive collaboration and active data sharing [1–3]. Projects such as recent organ and multi-organ single-cell atlas efforts [1, 4], Tabula Sapiens [5], and tissue-specific consortia [6] have generated comprehensive references covering most major organs and cell types. These resources have transformed how individual researchers annotate their own datasets: rather than relying solely on unsupervised clustering and manual marker inspection, investigators can now leverage reference-based annotation tools that transfer labels from well-curated atlases to new data. Resources and transfer-learning frameworks such as cross-tissue immune atlases, scArches, and Azimuth [7–9] have made this process accessible, providing pretrained models that enable rapid and reproducible cell type assignment even for non-expert users. This shift toward reference-based annotation represents a major advance in the standardization and scalability of single-cell analysis.

Importantly, these annotation tools already generate probability distributions over candidate cell types as an intermediate computational step, yet current workflows routinely discard this information after extracting the top-ranked label. Current annotation frameworks—ranging from correlation-based and probabilistic classifiers to deep learning-based or consensus-driven models—generate metrics to assess the reliability of cell-type assignments. Despite their technical diversity, these metrics are almost exclusively used to filter out low-confidence cells or to detect unassigned populations that do not match the reference. Consequently, the rich information embedded in annotation uncertainty remains largely underutilized as a technical artifact [10–14]. In each case, the uncertainty or confidence metric is treated as a quality-control filter—cells below a threshold are flagged for manual review or removed—rather than as a quantitative biological readout. This paradigm implicitly assumes that annotation uncertainty reflects technical failure (poor data quality, batch effects, or reference mismatch) rather than genuine biological signal.

In practice, however, reference-based annotation does not always produce clean results [15, 16]. A substantial fraction of cells in any given dataset may fail to match reference labels confidently: they receive low assignment scores, show ambiguous probability distributions across multiple cell types, or exhibit high entropy across the label space. Yet as atlas coverage has expanded to include most tissues and developmental stages [17], attributing these results strictly to technical factors is insufficient. If the reference is comprehensive and the data quality is adequate, what explains these persistently uncertain cells? We hypothesized that, in perturbation and disease-progression studies, the predominant contexts of single-cell analysis, annotation uncertainty itself may carry biologically meaningful information about identity destabilization, rather than simply reflecting technical failure.

Several computational frameworks have been developed to quantify cellular responses to perturbation, but none leverages annotation probability distributions as a biological signal. Existing computational frameworks quantify perturbation responses through distinct but complementary strategies: classifier-based cell-type separability [18], neighborhood-level compositional shifts [19], and mixture modeling of CRISPR perturbation signatures [20]. Recent efforts have also adapted energy-based and ensemble methods from computer vision for single-cell OOD detection [21]. However, these approaches share a fundamental limitation: they either operate at the population rather than single-cell level, require paired conditions or biological replicates, depend on RNA velocity or network priors, or treat OOD cells as annotation failures to be excluded rather than as biologically informative entities [22–24]. Critically, no existing framework systematically exploits the full annotation probability distribution—its entropy, shape, and multivariate structure—as a direct, continuous measure of cellular abnormality.

Single-cell transcriptomics is most frequently applied in perturbation settings, including genetic knockouts, drug treatments, and disease models, where the primary objective is to characterize how cellular identities destabilize rather than how faithfully they preserve canonical labels. In cancer biology, e.g. drug-tolerant persister (DTP) cells undergo reversible chromatin-mediated identity shifts that enable survival under therapeutic pressure [25], while large-scale CRISPR screens coupled with single-cell readouts (Perturb-seq) have revealed that genetic perturbations often produce graded, cell-type-specific transcriptional responses rather than discrete new states [26, 27]. In neurodevelopment, *in vivo* perturbation atlases have demonstrated that loss of transcription factors such as *Fezf2* destabilizes projection neuron identity without generating a distinct mutant-specific population [28]. In these contexts, cells often occupy transitional or continuum states that do not map cleanly onto predefined reference categories, and conventional differential expression or differential abundance analyses capture only part of this destabilization. We therefore reasoned that annotation uncertainty can itself carry biological signal: instead of treating low-confidence assignments as technical noise, they can mark biologically meaningful deviation from normative reference states. Accordingly, we interpret the full annotation probability vector—not a single best-match label, as a quantitative readout of identity instability and cellular deviation.

Here, we introduce AnnQ, a framework designed to quantify cellular deviation from a reference population in perturbation and disease-progression settings by treating annotation probability distributions as a quantitative biological readout rather than a technical byproduct. AnnQ derives uncertainty-aware features, assignment confidence, confidence gap, admixture ratio, and normalized entropy, from the full class-probability output and generates two complementary biological readouts of identity instability: (i) a discrete classification into four interpretable states, G0 (single stable identity), G1 (ambiguous or destabilized), G2 (within-lineage multi-identity), and G3 (cross-lineage multi-identity), each capturing a distinct mode of annotation instability; and (ii) a continuous out-of-reference (OOR) score computed via a covariance-aware distance metric relative to reference cells, such as wild-type or control populations. AnnQ detects aberrant and transitional states that are only weakly resolved by conventional clustering or differential-abundance analysis, thereby providing an interpretable quantitative layer for single-cell perturbation analysis.

Specifically, AnnQ makes three key contributions:

(1) It transforms annotation probability distributions into multivariate uncertainty features that capture identity instability.
(2) It introduces a continuous deviation metric, OOR score, to quantify cellular abnormality relative to normative reference states.
(3) It provides a perturbation-aware interpretative framework that links annotation instability to biological identity collapse and transitional cell states.

## Materials and methods

### Overview of the AnnQ framework

AnnQ is a reference-based framework designed to quantify cellular deviation using probabilistic cell type annotations. Instead of relying solely on discrete assignments, AnnQ uses probability distributions to model uncertainty in cell identity and measures deviation from a reference distribution. The framework consists of two main components: (i) uncertainty-aware cell state classification and (ii) cluster-conditional OOR deviation analysis.

### Input data and probabilistic annotation

AnnQ operates on a probability matrix generated by external annotation tools, such as CellTypist, where each cell is associated with a probability distribution over candidate cell types. Let ${P}_{i,j}$ denote the predicted probability that cell $i$ belongs to cell type $j$, with ${\sum}_j{P}_{i,j}=1$.

The input matrix is accompanied by cell-level metadata (e.g. sample, genotype, cluster assignment), which are not used for classification itself but are leveraged for stratified analyses and visualization.

### Uncertainty-aware feature representation

For each cell, AnnQ derives a compact set of uncertainty-aware features from the probability distribution:

- Assurance (P1): the highest predicted probability,
  $$P{1}_i=\underset{j}{\max }{P}_{i,j}$$
- Second-best probability (P2): the second highest predicted probability.
- Delta: the confidence gap between the top two predictions,
  $${\Delta}_i=P{1}_i-P{2}_i$$
- Admixture ratio: the relative strength of the second-best identity,
  $${A}_i=\frac{P{2}_i}{P{1}_i}$$
- Normalized entropy: a measure of global uncertainty across all cell types,
  $${H}_i=-\frac{1}{\log K}\sum_{j=1}^K{P}_{i,j}\log{P}_{i,j}$$

where $K$is the number of candidate cell types.

These features collectively quantify annotation confidence and identity dispersion.

### Cell state classification using threshold-based logic

Cells are classified into discrete AnnQ groups based on the number and strength of probability “hits” exceeding user-defined thresholds. A hard threshold (${T}_h$) defines confident assignments, while an optional soft threshold (${T}_s$) captures weaker but biologically meaningful signals.

For each cell, AnnQ counts how many cell type probabilities exceed the threshold and evaluates whether the corresponding cell types belong to the same or different hierarchical categories (tissue and cell class). Based on these criteria, cells are assigned to the following groups:

- G0: Single identity. Exactly one cell type exceeds the hard threshold, indicating a confident, unambiguous assignment.
- G1: Ambiguous or low-confidence. No cell type exceeds the hard threshold, indicating either uniformly low confidence or diffuse probability distribution across many types.
- G2: Multiple identities within the same lineage. Two or more cell types exceed the threshold, but all belong to the same tissue and cell class in the hierarchy. This pattern suggests biologically related identities, such as subtypes within a lineage.
- G3: Multiple identities across different lineages. Two or more cell types exceed the threshold, and they span different tissues or cell classes.

When soft thresholds are enabled, analogous groups (G2s, G3s) are assigned for cells where multiple types exceed ${T}_s$ but not ${T}_h$, capturing weaker multi-identity signals. To accommodate datasets with heterogeneous confidence profiles, AnnQ supports an adaptive mode in which thresholds are automatically suggested based on the interquartile range of the top probability (P1) distribution.

### Construction of biological reference distributions

To quantify cellular abnormality, AnnQ defines a reference distribution using wild-type or control cells. Rather than relying on a single global reference, AnnQ supports cluster-conditional reference stratification to control for intrinsic heterogeneity across cell types or contexts.

For each cluster $c$, if sufficient reference cells are available, AnnQ estimates a multivariate mean vector ${\mu}_c$and covariance matrix ${\Sigma}_c$using the uncertainty-aware features. Otherwise, a global reference distribution is used.

### Out-of-reference score calculation

Cellular deviation is quantified using the squared Mahalanobis distance:


$${\mathrm{OOR}}_i={\left({x}_i-{\mu}_c\right)}^T{\Sigma}_k^{-1}\left({x}_i-{\mu}_c\right)$$

where ${x}_i$ is the feature vector of cell i, and ${\mu}_c$ and ${\Sigma}_k$ denote the reference mean vector and covariance matrix for cluster k. We selected the Mahalanobis distance to account for covariance structure among uncertainty features, as these variables are inherently correlated and differ in scale. To ensure numerical stability, ${\Sigma}_k$ is regularized by shrinkage toward a diagonal covariance structure when cluster-level reference sample size is limited. Specifically, a shrinkage coefficient of λ = 0.1 was applied, balancing empirical covariance with its diagonal approximation. For clusters with fewer than *n* = 50 reference cells, covariance estimation was deemed unreliable, and a global reference covariance matrix was used as fallback.

This formulation accounts for correlations among uncertainty features and provides a scale-invariant measure of deviation.

### Out-of-reference summary statistics and tail enrichment

In addition to cell-level OOR scores, AnnQ computes cluster-level summary statistics, including:

- Median OOR shift: The difference in median OOR scores between conditions.
- Tail enrichment: The fraction of non-reference cells exceeding a specified quantile (e.g. 90th percentile) of the reference OOR distribution.

These summaries enable robust comparison across clusters and experimental conditions.

For statistical interpretation, the reference (e.g. wild-type) OOR distribution is treated as an empirical null model. Tail enrichment significance can be evaluated by estimating the expected exceedance rate under this null and applying either a binomial test or permutation-based resampling within clusters. Resulting *P*-values are adjusted for multiple testing using the Benjamini–Hochberg false discovery rate procedure across clusters. This framework provides a principled assessment of whether observed OOR shifts exceed reference-derived variability.

### Implementation and availability

AnnQ is implemented in Python 3.9+. The package provides both a command-line interface and a Python API for integration into existing single-cell analysis workflows. AnnQ accepts probability matrices from standard annotation tools including CellTypist, and outputs classification results, OOR scores, and summary statistics as CSV files. Visualization functions generate publication-ready plots for quality control and result interpretation. Configuration is handled via YAML files, with default parameters and example configurations provided in the documentation. Source code, tutorials, and example datasets are available at https://github.com/joonan-lab/AnnQ.git. AnnQ is released under the MIT license.

## Results

### Overview of the AnnQ package

**Figure 1:**
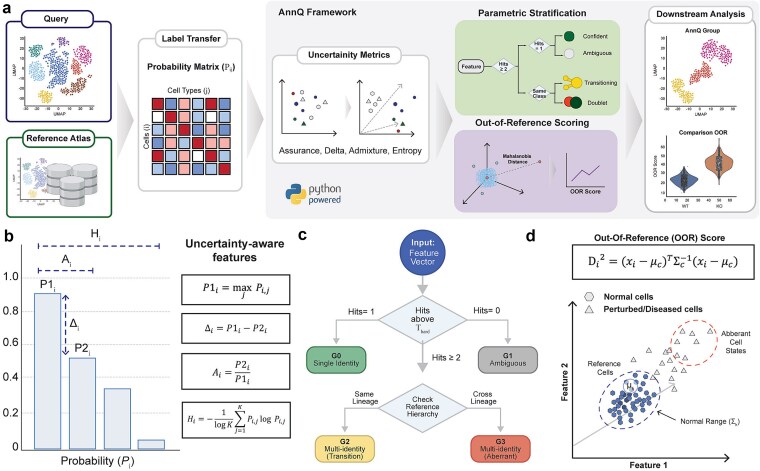
Overview of the AnnQ package. (a) Flowchart illustrating the sequential steps of AnnQ analysis, starting from probabilistic cell type assignments to feature extraction, group classification, and OOR score calculation. (b) Uncertainty-aware feature metrics. Representative plots of the four primary features derived from the probability distribution: Assurance (P1), Confidence gap (Delta), Admixture ratio, and Normalized entropy. Each metric illustrates a different dimension of the probabilistic mapping for an individual cell. (c) Threshold-based classification into AnnQ groups. Diagram showing the logic for assigning cells into four discrete categories based on probability thresholds: G0 (Single identity), G1 (Ambiguous/Low confidence), G2 (Multi-identity within the same lineage), and G3 (Multi-identity across different lineages). (d) Calculation of OOR scores. Schematic showing the projection of query cells onto a multivariate uncertainty space defined by a reference population. The OOR score is represented as the squared Mahalanobis distance from the reference centroid, indicated by the distance vector between the query point and the reference distribution. Created with Biorender.com

AnnQ is a Python package that quantifies cellular abnormality by transforming probabilistic annotation outputs into a multivariate uncertainty space and measuring deviation from a reference population (Fig. 1a). The package takes as input a probability matrix from reference-based annotation tools such as CellTypist [7], where each cell is assigned a distribution over candidate cell types. From this distribution, AnnQ extracts four uncertainty features capturing complementary aspects of annotation confidence: assurance (P1), defined as the maximum probability; confidence gap (delta), the difference between the top two probabilities; admixture ratio, the relative strength of the second-best identity; and normalized entropy, measuring dispersion across all candidates (Fig. 1b). These features together define a multivariate uncertainty space in which each cell occupies a position reflecting not just its most likely identity, but also the structure of its annotation ambiguity.

Using these features, AnnQ classifies cells into discrete groups based on threshold logic applied to the probability distribution (Fig. 1c). G0 cells have exactly one cell type exceeding the confidence threshold, indicating a single clear identity. G1 cells have no cell type exceeding the threshold, reflecting low confidence or diffuse probability mass. G2 and G3 cells have multiple cell types exceeding the threshold, distinguished by whether those types belong to the same hierarchical lineage (e.g. subtypes within T cells) or span different lineages (e.g. epithelial and mesenchymal). This classification provides an interpretable categorical summary of annotation certainty that can be used to flag biologically interesting populations for further investigation. These group assignments are robust to common technical covariates: QC metric distributions (nGene, nUMI, percent mitochondrial reads, and Scrublet doublet score) overlap substantially across groups, AnnQ group proportions remain stable under a range of QC and doublet filtering scenarios, and AnnQ uncertainty features show only weak correlations with these metrics (Supplementary Fig. S1). Beyond technical covariates, AnnQ classifications were stable against reference-label ambiguity in two complementary tests: a counterfactual probability-splitting experiment preserved >99% of G0 assignments across α = 0.5–0.9 split ratios, and the most frequent G2/G2s competing-label pairs observed in real data (e.g. basal vs. LumSec-basal, CD4-activated vs. CD4-Treg) aligned with known biological continua rather than redundant subtype labels (Supplementary Fig. S1D–F).

Beyond discrete classification, AnnQ computes a continuous OOR score measuring each cell’s deviation from a reference population in uncertainty feature space (Fig. 1d). The reference is defined using control cells—typically wild-type or untreated samples—from which AnnQ estimates a multivariate mean and covariance matrix. For each cell, deviation is calculated as the squared Mahalanobis distance from this reference distribution, a formulation that accounts for correlations among uncertainty features and provides a scale-invariant measure. OOR scores can be computed globally or stratified by cluster to control for intrinsic heterogeneity across cell types, with automatic fallback to global reference when cluster-specific reference cells are insufficient. The package supports both command-line execution via YAML configuration files and direct integration into Python workflows, with visualization functions for quality control and result interpretation.

### Atlas-scale application of AnnQ across the Mouse Brain Perturbation Atlas

**Figure 2:**
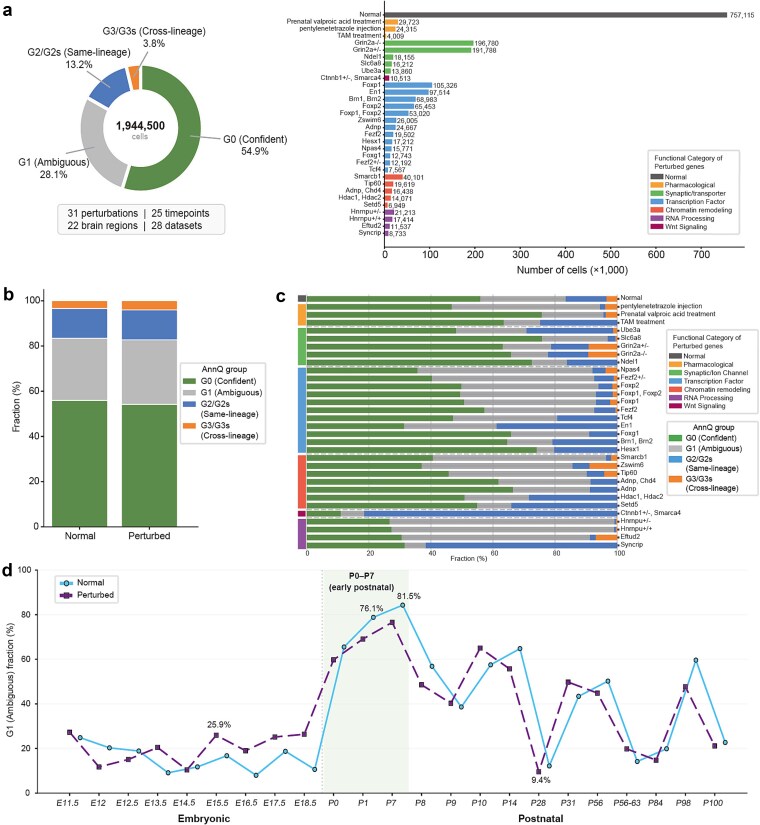
Atlas-scale application of AnnQ across the Mouse Brain Perturbation Atlas. (a) AnnQ classification of 1 944 500 cells across the Mouse Brain Perturbation Atlas encompassing 31 perturbations, 25 timepoints, 22 brain regions, and 28 datasets. Left: donut chart showing the overall distribution of AnnQ groups. Right: horizontal bar chart displaying cell counts per perturbation, colored by functional category. Annotation was performed using the CellTypist Developing Mouse Brain model. (b) Stacked bar plots comparing AnnQ group composition between Normal (wild-type) and Perturbed conditions. G0, G1, G2/G2s, and G3/G3s fractions are shown for each condition. (c) Perturbation-level AnnQ group fractions. Left color bars indicate the functional category of each perturbation (RNA processing, chromatin remodeling, transcription factor, Wnt/signaling, synaptic/transporter, pharmacological, and normal). Right stacked bars show the relative proportion of each AnnQ group per perturbation. (d) G1 fraction plotted across developmental stages from embryonic (E11.5–E18.5) to postnatal (P0–P100) timepoints for Normal (green) and Perturbed (red) conditions.

To assess the scalability of AnnQ and to characterize annotation uncertainty across diverse genetic perturbations, we applied the framework to the Mouse Brain Perturbation Atlas, comprising 1 44 500 cells across 24 perturbations, multiple developmental stages (E14.5–P28), and diverse brain regions. AnnQ classified the full atlas by employing stage-specific CellTypist models: the Developing Mouse Brain model [29] for embryonic stages and the Whole Mouse Brain model for postnatal stages [30]. Through this approach, 54.9% of cells were assigned to G0, 28.1% to G1, 13.2% to G2/G2s, and 3.8% to G3/G3s (Fig. 2a, Table S1). [28, 31–57]

A global comparison of AnnQ group compositions revealed that perturbed populations exhibit a systemic shift toward identity instability, characterized by an expansion of the G1 and G3/G3s fractions relative to normal controls (Fig. 2b). AnnQ revealed distinct heterogeneity in annotation uncertainty across perturbations, with G1 fractions ranging from 5.5% (*Hesx1*) to 72.4% (*Hnrnpu* heterozygous). *Hnrnpu*, a core ribonucleoprotein essential for global RNA processing and splicing [58], exhibited the highest G1 fraction, consistent with its fundamental role in maintaining transcriptomic architecture. Other high-uncertainty perturbations included *Eftud2* (60.6%, spliceosome component) and *Smarcb1* (55.7%, SWI/SNF chromatin remodeling), suggesting that disruptions at the RNA processing or chromatin level produce the most profound destabilization of cell identity.

In contrast, *Ctnnb1* (Het) and *Smarca4* displayed low G1 fractions (7.6%) but the highest G2 fractions in the atlas (81.5%), indicating retention of multi-identity states within the same developmental lineage (Fig. 2c). *Ctnnb1* encodes β-catenin, a central Wnt pathway effector governing fate decisions between closely related cortical subtypes [59, 60].

A notable finding was the extreme annotation uncertainty in early postnatal cells (P0–P7), with G1 fractions reaching 62.8% at P0, 76.1% at P1, and 90.4% at P4—far exceeding embryonic (9.0%–14.0%) and mature (9.4% at P28) stages (Fig. 2d). This period is well established as a window of profound cellular transformation involving rapid neuronal maturation, synaptogenesis, and gliogenesis [29, 61], placing cells in a genuinely transitional state that lacks a single definitive transcriptomic identity. The elevated G1 fractions thus reflect a biological signature of developmental plasticity rather than technical noise.

We performed four semi-simulation analyses to validate that this G1 enrichment reflects a genuine biological signal rather than artifacts of the reference configuration, using one of the perturbation datasets from the Mouse Brain Perturbation Atlas as a representative case. For each analysis, the Di Bella 2021 E15.5 cortical reference was systematically modified while holding the query data fixed, allowing controlled perturbation of reference properties in isolation [28]. In a reference-lineage dropout experiment, systematically withholding individual cell types from the reference revealed that cells with a well-defined transcriptional identity, including CThPN (99.9%), Interneurons (99.6%), and subcerebral projection neuron (SCPN) (94.9%), were captured as G1 upon dropout (Supplementary Fig. S2A). Sweeping reference coverage from 30% to 100% showed that ΔG1(Hom − Het) remained consistently elevated across all levels (+12.2 to +18.2 percentage points), ruling out reference sparsity as a confound (Supplementary Fig. S2B). Progressive coarsening of cell-type labels from 14 fine-grained subtypes to six broader classes maintained a consistent ΔG1 elevation (Supplementary Fig. S2C). Applying AnnQ with five independent cortical atlases as alternative references consistently yielded higher G1 in Hom compared to Het (ΔG1 = +1.8 to +12.8 pp across all five references), establishing cross-reference reproducibility of the homozygous identity instability phenotype (Supplementary Fig. S2D) [39, 47, 50, 54, 56]. These results confirms the robustness of the G1 signal as a quantitative readout of cellular perturbation.

### Cross-atlas validation and discovery across five independent datasets

**Figure 3:**
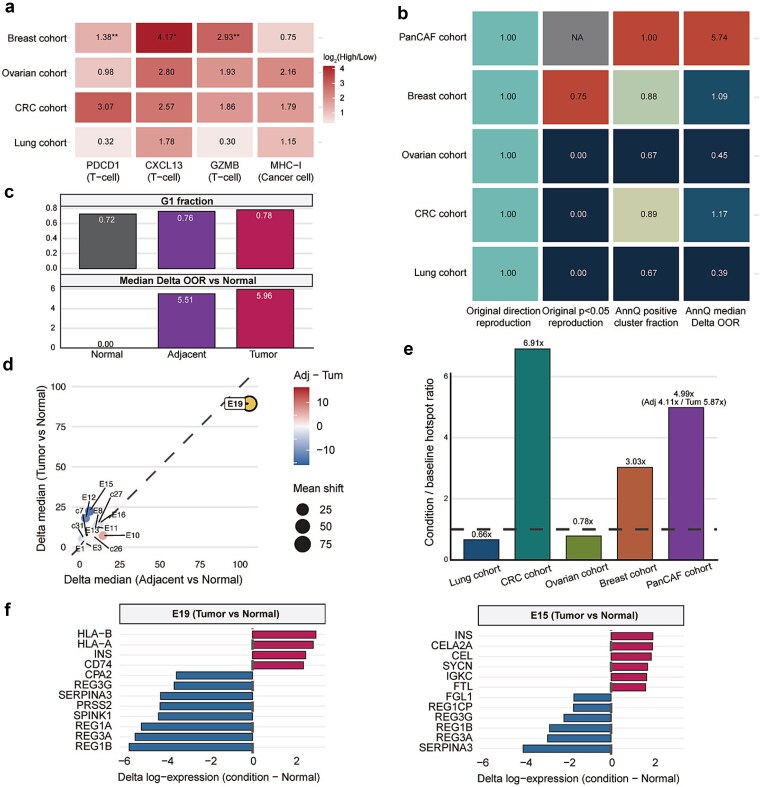
Cross-atlas AnnQ validation and discovery across five independent datasets. (a) Reproduction of exhaustion-associated gene axes across four Lambrechts cohorts (*PDCD1, CXCL13, GZMB, MHC-I* score; HighExh vs. LowExh). Each cell in the heatmap represents the log2(HighExh/LowExh) value. (b) Side-by-side comparison of original analysis and AnnQ reproduction for each dataset. Metrics include direction reproduction, original *P* < .05 reproduction, AnnQ positive fraction, and AnnQ median delta OOR. (c) Reproduction of the PanCAF normal-to-lesion axis: G1 fraction and median delta OOR. Both G1 fraction and OOR deviation increase progressively from Normal to Adjacent to Tumor. (d) Asymmetric remodeling between Adjacent and Tumor states in PanCAF. E19 is highlighted as the strongest shared-shift cluster. The x-axis represents the Adjacent vs. Normal delta median, and the y-axis represents the Tumor vs. Normal delta median. (e) OOR hotspot enrichment ratio, defined as the condition-to-baseline ratio, quantifying the extent to which top OOR cells are enriched in the condition state across each dataset. (f) Compact DEG view of the top OOR epithelial cluster. Delta log expression values are shown for E19 (Adjacent vs. Normal) and E19 (Tumor vs. Normal).

To evaluate whether AnnQ generalizes beyond the primary atlas-scale case studies, we expanded the analysis to five additional datasets: four independent Lambrechts cohorts (lung, colorectal, ovarian, and breast cancers) and one pan-cancer fibroblast atlas (PanCAF). For the Lambrechts cohorts, we stratified patients into HighExh and LowExh groups using T-cell exhaustion markers and quantified condition-specific deviation using cluster-level OOR shifts relative to the LowExh baseline. At the gene level, exhaustion-associated marker expression (*PDCD1, CXCL13, GZMB*, and *MHC-I* score) showed positive log_2_ fold changes in HighExh vs. LowExh across all four cohorts, with particularly strong signals in CRC and Breast cancers (Fig. 3a). Translating these findings into AnnQ’s annotation uncertainty framework, we observed positive median OOR shifts in HighExh vs. LowExh (CRC: 1.17, Breast: 1.09, Ovarian: 0.45, Lung: 0.39), with CRC and Breast showing the strongest and most consistent effects (Fig. 3b, Supplementary Fig. S3). AnnQ-derived metrics showed high concordance with findings from original publications, reproducing the directionality of established disease-associated axes across all cohorts. Median delta OOR values aligned with previously reported statistical significance thresholds, mirroring the biological signals identified through conventional differential expression and pathway enrichment analyses. In the counts-based PanCAF reanalysis (Normal baseline vs. Adjacent/Tumor), all tested cluster-condition comparisons showed positive OOR median shifts (95/95), and the global G1 fraction increased from 0.7237 in Normal to 0.7594 in Adjacent and 0.7814 in Tumor (Fig. 3c). Importantly, shift magnitude was not monotonic across all clusters: some epithelial programs were stronger in Adjacent (e.g. E19, E10), whereas others were stronger in Tumor (e.g. E15, E12), revealing parallel field-effect and tumor-core remodeling axes that are not captured by discrete annotation labels alone (Fig. 3d). To assess the spatial distribution of annotation uncertainty, we constructed single-cell UMAP manifolds from AnnQ probability vectors and quantified the enrichment of high-OOR cells within condition-specific vs. baseline populations (Supplementary Figs S4 and S5). Elevated OOR cells localized to specific subregions rather than appearing uniformly across the map, indicating focal instability hotspots within otherwise continuous cell-state structures. The OOR hotspot enrichment ratio, defined as the condition-to-baseline ratio of top-decile OOR cells, confirmed that these high-uncertainty states are systematically enriched in disease conditions across all datasets (Fig. 3e).

At the gene level, the top shared-shift cluster E19 showed a lesion-associated axis with concordant upregulation of antigen-presentation and immune-interaction genes (*HLA-A, HLA-B, HLA-C, CD74, CCL5, CXCR4*) in both Adjacent and Tumor vs. Normal. In contrast, an exocrine-like program (*PRSS1, CPA1, CLPS, AMY2A*) showed stronger depletion in Tumor than in Adjacent, quantitatively matching the non-monotonic Adjacent–Tumor split seen in OOR space (Fig. 3f; Supplementary Fig. S6). Extending this analysis to all top OOR clusters (E17/E20/E19 in Adjacent and E19/E15/E12 in Tumor) revealed distinct cluster-specific DEG architectures rather than a single shared response: pancreatic exocrine-like suppression (*REG1A/REG1B/REG3A/SERPINA3*), epithelial antigen-presentation activation (*HLA-A/B/C* and *CD74*), and context-specific secretory programs varied across clusters and lesion states (Supplementary Fig. S7). Together, these analyses support both reproducibility of known disease-associated instability patterns and discovery of previously unappreciated multi-axis deviation structure across independent atlases.

### Case study 1: AnnQ quantifies aberrant neuronal states in a Fezf2 perturbation model

**Figure 4:**
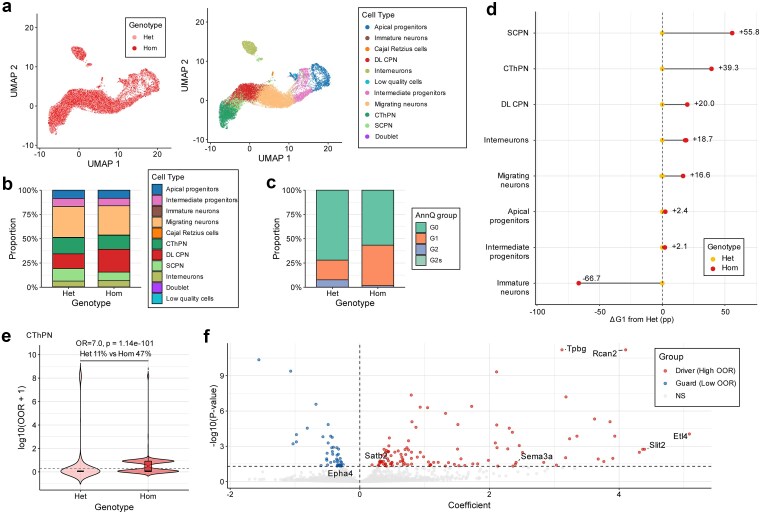
AnnQ quantifies aberrant neuronal states in a *Fezf2* perturbation model. (a) UMAP embedding of E15.5 cortical cells colored by genotype (left) and AnnQ-predicted cell type (right). (b) Stacked bar plots showing the composition of predicted cell types. (c) Stacked bar plots showing the composition of AnnQ groups across genotypes. (d) Dumbbell plot showing per-cell-type G1 fraction shift between Het (baseline, yellow) and Hom (red). The x-axis represents ΔG1 from Het (percentage points); left-side labels indicate the absolute G1 rate in Het. (e) Violin plots displaying the distribution of OOR scores for CThPN (log_₁₀_ scale). Statistical significance was assessed using Fisher’s exact test on the OOR > 1 tail (Het 11.1% vs. Hom 46.9%; OR = 7.05, *P* = 1.14 × 10^−101^). (f) Volcano plot of log-linear regression coefficients identifying molecular drivers of OOR scores in the CThPN population.

Building on the broad patterns observed in the atlas-scale analysis, we next sought to validate AnnQ’s ability to resolve nuanced, biologically driven aberrant states within a specific genetic context. To this end, we applied the framework to a single-cell RNA-seq dataset from a *Fezf2* perturbation model of cortical neuron development [28]. In this study, loss of *Fezf2* disrupted canonical SCPN identity and gave rise to aberrant corticothalamic-like neuronal states, without forming a clearly separable new population. Specifically at E15.5, the perturbation primarily resulted in a destabilization of the Corticofugal Projection Neuron (CFuPN) lineage, biasing cell identities toward Cortical Projecting Neuron (CPN)- or Corticothalamic Projection Neuron (CThPN)-like states, with this shift most pronounced in homozygous (Hom) knockout compared to heterozygous (Het) controls. Despite significant transcriptional perturbation, standard integration and clustering showed a largely preserved global landscape without emergence of a distinct homozygous-specific cluster (Fig. 4a). Likewise, predicted cell-type proportions changed only modestly (Fig. 4b), indicating that conventional clustering and differential-abundance analyses captured composition shifts but not the degree of identity destabilization.

We therefore analyzed uncertainty structure rather than relying only on maximum-probability labels. *Fezf2* homozygous mutants exhibited a ~ 2.1-fold expansion of the ambiguous (G1) population relative to Het (Hom 41.8% vs. Het 20.4%; Fig. 4c) and per-cell-type G1 analysis revealed that the largest Hom-vs.-Het G1 increases occurred in SCPN (+55.8 pp), CThPN (+39.3 pp), DL CPN (+20.0 pp), and Migrating neurons (+16.6 pp), confirming the selective destabilization of the CFuPN lineage (Fig. 4d). The emergence of a prominent high-OOR tail demonstrates that this annotation uncertainty reflects a biologically driven aberrant state rather than mere technical noise (Fig. 4e). The OOR tail enrichment in homozygous CThPN cells was significant (Fisher’s exact test on OOR > 1: Het 11.1% vs. Hom 46.9%, OR = 7.05, *P* = 1.14 × 10^−101^), consistent with *Fezf2*-dependent disruption of neuronal identity. Furthermore, gene-level log-linear regression of OOR on expression (controlling for the number of genes and number of feature counts) across Het and Hom CThPN cells combined (*n* = 2783) identified *Satb2, Slit2*, and *Sema3a* as significant contributors to elevated OOR, further confirming the biological relevance of these structurally dysregulated states (Fig. 4f).

### Case study 2: AnnQ quantifies drug-tolerant and metastatic precursor states in breast cancer

To evaluate AnnQ in a context of dynamic cellular plasticity, we also applied the framework to a longitudinal single-cell RNA-seq dataset from a stage IV breast cancer patient [62]. Samples were collected before treatment (Pre), after chemotherapy from two primary tumor regions (Post1, Post2), and from a distant peritoneal metastasis (Meta). The original study identified DTP cells [25] and metastatic precursor cells (MPCs) by integrating transcriptomics with copy number variation inference. We investigated whether AnnQ could characterize these transient states solely through probabilistic annotation dynamics.

**Figure 5:**
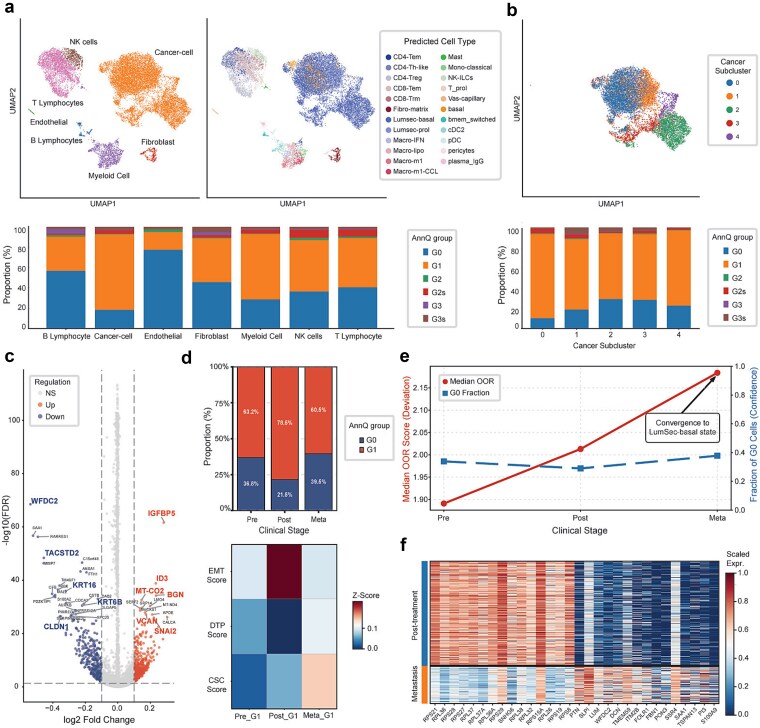
AnnQ quantifies drug-tolerant and metastatic precursor states in breast cancer. (a) UMAP visualization of expert-annotated labels (top left) and CellTypist predicted labels (top right). The stacked bar plot (bottom) represents the relative proportion of AnnQ groups (G0–G3) within each expert-annotated cell type. (b) UMAP of cancer-specific sub-clusters (top) and their internal AnnQ group proportion revealing transcriptional heterogeneity within the misannotated population (bottom). (c) Volcano plot of DEGs between G0 and G1 populations. (d) Stacked bar chart of G0 and G1 proportions across clinical stages (Pre, Post, Meta) (top) and a heatmap showing the dynamics of EMT, DTP, and CSC scores within G1 cells during metastatic progression (bottom). (e) Dual-axis line plot displaying the median OOR score (left y-axis, red) calculated by Mahalanobis distance and the fraction of G0 cells (right y-axis, blue) across stages. (f) Heatmap of standard-scaled (0 to 1) expression levels for top DEGs in cancer clusters 4 and 13, comparing post-treatment and metastatic samples.

A fundamental challenge in reference-based annotation is the “forced classification” of novel states into existing categories [21]. Since the reference atlas lacked a “malignant” label, cancer cells were inevitably assigned to the “Luminal cell” lineage (Fig. 5a). This forced alignment masks transcriptional deviation because probabilities are calculated based on shared features with normal cells while disregarding cancer-intrinsic aberrant features. Consequently, cancer cells display uncertainty patterns statistically similar to the healthy baseline, leading to an underestimation of the OOR score.

To overcome this, we used AnnQ’s uncertainty metrics to uncover hidden heterogeneity within these forced labels. By stratifying cells into G0 and G1 groups, we observed spatial segregation in the UMAP embedding and distinct compositional differences in subcluster analysis (Fig. 5b). Differential expression analysis confirmed that while G0 cells retained canonical luminal markers, G1 cells exhibited a loss of epithelial features alongside significant upregulation of Epithelial–Mesenchymal Transition (EMT) drivers (e.g. *SNAI2*) and stress-response genes (Fig. 5c).

We next hypothesized that G1 dynamics would mirror the tumor’s evolutionary trajectory. In the pre-treatment sample, AnnQ detected that 63.2% of the G1 population expressed a weak DTP signature, whereas the G1 fraction expanded by 78.5% in post-treatment (Fig. 5d). These cells showed a sharp increase in EMT scores [63–66] and concurrent downregulation of the DTP program [67–73], signaling a transition into proliferative MPCs corresponding to these stages. In metastasis (Meta-G1), we observed a reactivation of cancer stem cell (CSC) markers and reduced EMT scores, reflecting the mesenchymal–epithelial transition required for tissue colonization [74–78].

Finally, we used the OOR score to quantify deviation from the healthy baseline. While OOR scores generally increased post-treatment, the metastatic population presented a paradox: deviation reached its peak, yet the proportion of G0 cells simultaneously increased compared to the post-treatment stage (Fig. 5e). This occurs because metastatic cells converge toward “LumSec-basal”, a reference state characterized by intermediate luminal-basal features. Since the reference model included this transitional state, metastatic cells were recognized with high assurance. Molecular evidence from top DEGs confirmed that this structural reorganization is not stochastic noise but a fundamental shift into a defined abnormal architecture (Fig. 5f).

## Discussion and conclusion

Existing single-cell annotation tools generate probability distributions over candidate cell types but routinely discard this distributional information after extracting the top-ranked label, treating annotation uncertainty as a quality-control problem rather than a biological signal. We developed AnnQ to address this gap: by retaining and analyzing the full annotation probability distribution, AnnQ transforms what was previously discarded metadata into a quantitative measure of cellular identity instability. This framing is supported by recent studies demonstrating that continuous, intermediate cell states are a defining feature of disease progression. For instance, colorectal cancer harbors a transcriptional continuum of stem-to-differentiated states that discrete labels fail to capture [79], and primary vs. metastatic breast tumors exhibit marked diversity in transitional cell states that similarly resist binary classification [80]. Across genetic perturbation and cancer progression case studies, AnnQ detected aberrant and transitional states that were not resolved by conventional clustering, differential abundance testing, or discrete label assignment—demonstrating that annotation uncertainty encodes biological information about cell state destabilization.

A key strength of AnnQ is detection of destabilized identities that do not form separable clusters. In *Fezf2* perturbation, standard clustering and differential abundance analyses were insufficient because mutant cells did not form a novel, isolated population detectable by population-level or compositional shifts alone. AnnQ instead revealed coordinated expansion of G1 cells with elevated OOR, consistent with collapse of canonical SCPN identity rather than emergence of a new stable type. The association of higher OOR with *Satb2* and axon-guidance genes (*Slit2, Sema3a*) further indicates that these cells represent biologically meaningful aberrant trajectories, not technical noise.

AnnQ provides a complementary perspective to existing perturbation quantification approaches. Population-level methods such as Augur [18] prioritize cell types that are most responsive to perturbation based on transcriptional separability, while differential abundance methods such as Milo [19] sensitively detect shifts in local cellular neighborhood composition, and entropy-based potency measures such as SCENT [24] capture differentiation potential through network-level signaling entropy. These approaches address distinct aspects of perturbation biology and remain valuable for their respective purposes. In contrast, AnnQ introduces an orthogonal axis of information by measuring deviation in annotation probability space rather than expression space. By retaining the full distribution of reference-based annotation probabilities, AnnQ quantifies how strongly an individual cell’s identity deviates from expected reference states, thereby providing a reference-aware measure of identity destabilization. This perspective captures aspects of cellular abnormality that are distinct from, yet complementary to, transcriptional distance or compositional abundance metrics, and can therefore be integrated with these approaches to provide a more complete view of perturbation-induced cellular change.

AnnQ also addresses forced classification in reference-based analysis. When cancer cells are mapped to healthy references, malignant states are often assigned to the nearest normal-like category (e.g. luminal), which can mask true pathological deviation. In the longitudinal breast cancer case study, discrete labels remained relatively stable, whereas uncertainty-derived metrics (G1 fraction and OOR) tracked disease-state transitions from DTPs toward metastatic precursor programs with high EMT signatures.

The metastatic paradox, defined by persistently high OOR despite recovery of confident G0 assignment, highlights the complementary nature of AnnQ’s discrete and continuous outputs. The G0–G3 classification reflects the modal property of each cell’s own probability distribution, whereas the OOR score quantifies the distance of each cell’s multivariate uncertainty profile from the empirical healthy/control baseline distribution, computed as a covariance-aware Mahalanobis distance against a cluster-conditional reference. Because annotation tools operate within a fixed reference space, pathological cells can still be mapped with high confidence to the closest known class. AnnQ adds a critical complementary measure: a confident modal label G0 can coexist with substantial deviation of the cell’s uncertainty profile from the WT baseline, and the OOR score preserves that signal. That said, the absolute magnitude of OOR can be influenced by reference composition and lineage-specific variance structures. To address this, relative deviation metrics, such as cluster-normalized OOR shifts or within-lineage delta scores, can serve as complementary indicators, enabling more context-aware interpretation of cellular identity reprogramming dynamics.

As a framework that derives its metrics from the annotation probability distribution, AnnQ is most reliable when applied to well-curated reference atlases. For study-specific contexts, users can intervene upstream at two explicit points within the workflow: a user-editable hierarchy JSON (for encoding custom label relationships or adding references) and the input probability matrix CSV (where known redundant columns can be merged before classification). In addition, expanded multimodal references and generative modeling approaches may further refine the boundaries of normative uncertainty.

By systematically quantifying deviation in annotation probability space, AnnQ establishes a general framework for extracting this signal and introduces a new analytical axis for studying cellular plasticity, instability, and disease-associated reprogramming at single-cell resolution.

## Supplementary Material

Supplementary_Material_bbag278

## Data Availability

The AnnQ framework is implemented in Python and is openly available at the GitHub repository (https://github.com/joonan-lab/AnnQ.git) under the MIT license. The Mouse Brain Perturbation Atlas dataset used for validation in this study is available via Zenodo (https://doi.org/10.5281/zenodo.17112344). All processed data and analysis scripts required to reproduce the figures are available in the GitHub repository. All other datasets used in the case studies are publicly available from their original publications and repositories.
