# Supplementary material for: AnnQ: reference-based quantification of cellular abnormality at single-cell resolution
Source: Brief Bioinform. 2026 May 31;27(3):bbag278. doi: 10.1093/bib/bbag278 (PMC13222523; doi:10.1093/bib/bbag278)
Supplement: Supplementary_Material_bbag278 [file supplementary_material_bbag278.zip › Supplementary_materials_Revised_bbag278(1).docx]

**AnnQ: reference-based quantification of cellular abnormality at single-cell resolution**

Davin Lee^1,2,3†^, Gaeun Byeon^1,3,4,†^, Seojin Chung^4^, Dongmin Shin^1,3,4^, Jongseo Park^6^, Ingyeong Koh^1,3,4,7,8^, Joon-Yong An^1,2,3,4,5,*^

^1^ Department of Integrated Biomedical and Life Science, Korea University, Seoul, 02841, Republic of Korea

^2^ Interdisciplinary Major Program in Targeted Degradation-based Innovative Therapeutics, Korea University, Seoul, 02841, Republic of Korea

^3^ National Research Laboratory for Convergence Degradation Biology, Korea University; Seoul, 02841, Republic of Korea

^4^ L-HOPE Program for Community-Based Total Learning Health Systems, Korea University, Seoul, 02841, Republic of Korea

^5^ School of Biosystem and Biomedical Science, College of Health Science, Korea University, Seoul, 02841, Republic of Korea

^6^ School of Health and Environmental Science, College of Health Science, Korea University, Seoul, 02841, Republic of Korea

^7^ Genetics and Genome Biology Program, The Hospital for Sick Children; Toronto, ON M5G 0A4, Canada

^8^ The Centre for Applied Genomics, The Hospital for Sick Children; Toronto, ON M5G 0A4, Canada

^†^ Joint Authors

^*^ Corresponding author

Dr. Joon-Yong An

Email: joonan30@korea.ac.kr; Phone: 82 2 3290 5646

**Supplementary Figures**

**
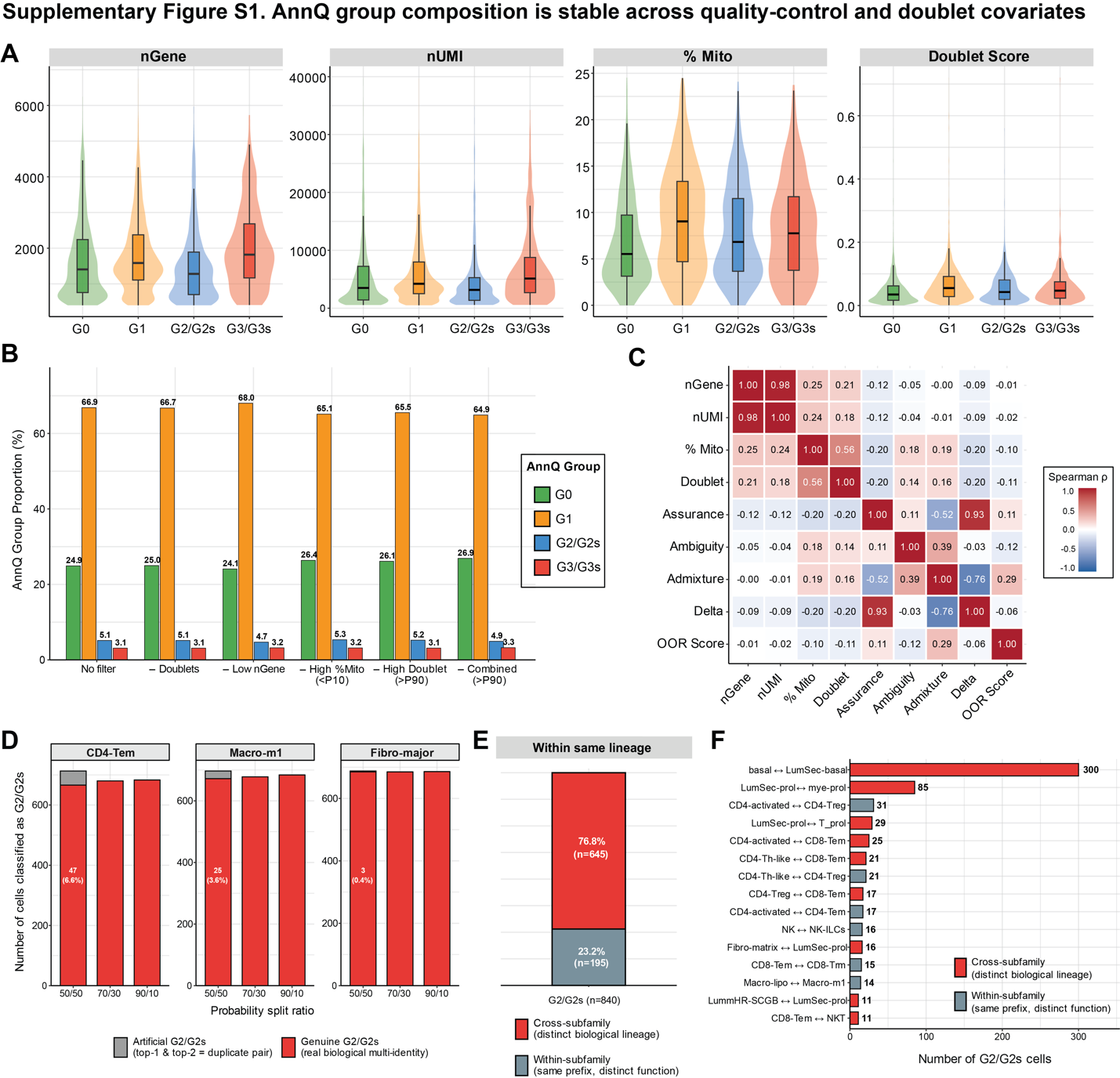
**

**Supplementary Figure S1.** AnnQ group composition is stable across quality-control and doublet covariates. Breast cancer case-study dataset (n = 16,350 cells). **(A)** QC metric distributions—nGene, nUMI, % Mito, and Scrublet doublet score—by AnnQ group (G0, G1, G2/G2s, G3/G3s). **(B)** AnnQ group proportions (%) after six filtering scenarios: no filter, removal of predicted doublets, nGene < P10, % Mito > P90, doublet score > P90, and all four combined. Each bar shows one group’s fraction of retained cells. **(C)** Pairwise Spearman correlations (ρ) among the four QC metrics and five AnnQ uncertainty features (Assurance, Ambiguity, Admixture, Delta, OOR score). **(D)** Counterfactual label-duplication simulation showing the proportion of newly classified G2/G2s cells attributable to artificial duplicate pairs versus genuine competing labels across subtypes and split ratios; artificial cases remain a minority even under extreme conditions. **(E)** Hierarchy-level classification of G2/G2s cells in the breast cancer case study, showing that while all competition occurs within Cell Classes, most cases span distinct subfamilies. **(F)** Top competing subtype pairs ranked by frequency, with dominant pairs corresponding to known biological continua rather than label redundancy.

Alt text: Six-panel supplementary figure showing that AnnQ group assignments are robust to quality-control metrics, doublet contamination and ambiguous label in the breast cancer dataset. A shows violin plots of nGene, nUMI, % Mito, and doublet score across the four AnnQ groups with overlapping distributions; B shows a grouped bar chart in which AnnQ group proportions remain nearly constant across six technical filtering scenarios; C shows a Spearman correlation heatmap between QC metrics and AnnQ uncertainty features; D shows the proportion of newly classified G2/G2s cells attributable to artificial duplicate pairs versus genuine competing labels across subtypes and split ratios; E shows a stacked bar plot of classification of G2/G2s cells in the breast cancer case study, showing that while all competition occurs within Cell Classes, most cases span distinct subfamilies; F shows a bar plot, showing top competing pairs in those of G2/G2s cells, with dominant pairs corresponding to known biological continua rather than label redundancy.

**
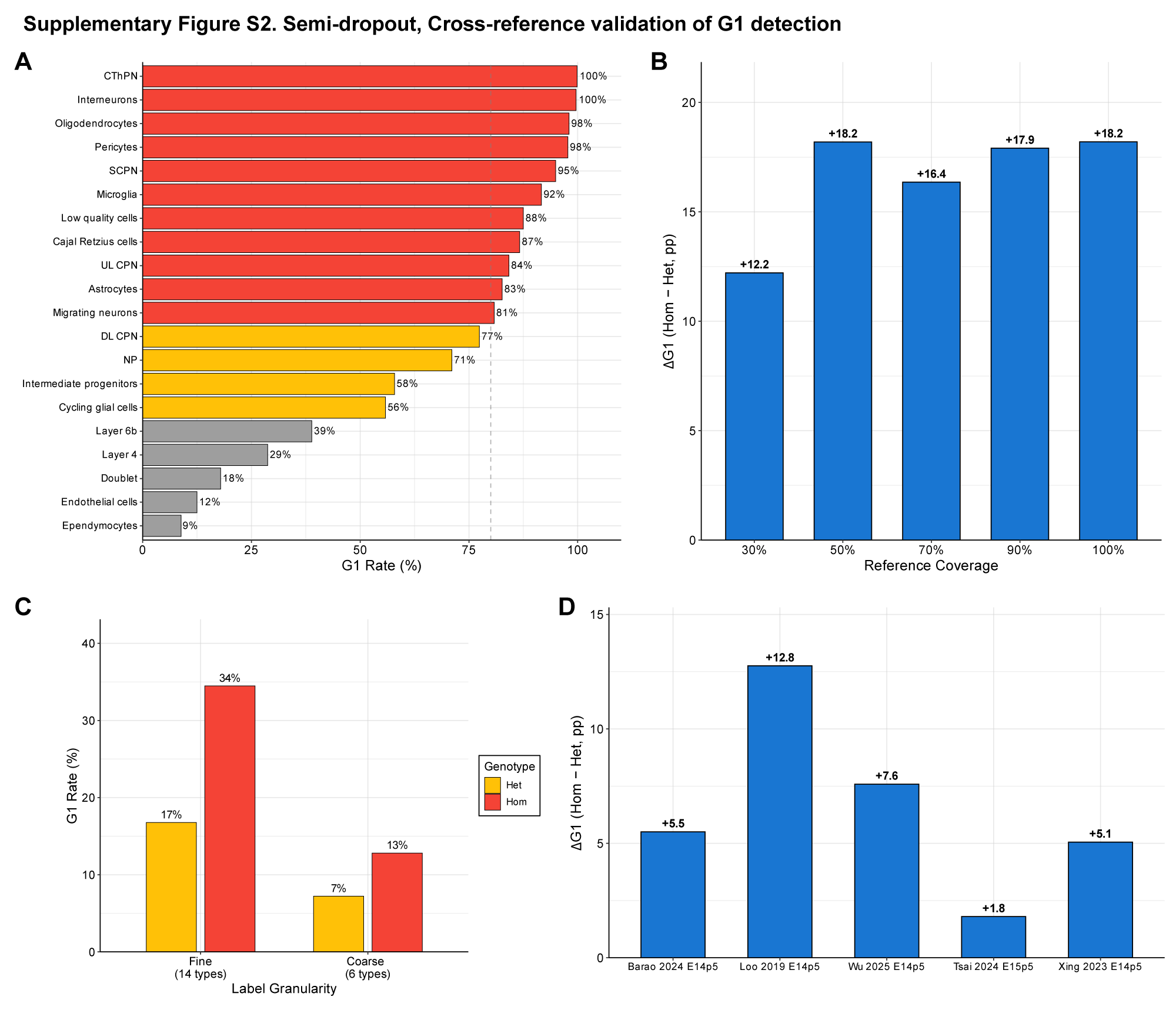
**

**Supplementary Figure S2.** **(A)** Barplot showing the G1 rate of held-out cells when each cell type in the Bella 2021 E15.5 cortical reference was systematically withheld and query cells of the corresponding type were re-annotated. Bars are sorted by G1 rate and colored by threshold (red ≥80%, yellow ≥50%, grey <50%). Dashed line indicates the 80% threshold. **(B)** Barplot showing ΔG1 (Hom − Het, percentage points) when the Bella 2021 reference was randomly downsampled to five coverage levels (30–100%) and AnnQ was re-applied to the full Het and Hom query cohorts. **(C)** Grouped barplot showing G1 rates for Het (yellow) and Hom (red) when reference annotations were coarsened from 14 fine-grained subtypes to 6 broader classes. **(D)** Barplot showing ΔG1 (Hom − Het, percentage points) when AnnQ was independently applied using five external cortical atlases as references (Barao 2024, Loo 2019, Wu 2025, Tsai 2024, Xing 2023).

Alt text: Four-panel supplementary figure showing that AnnQ-based G1 enrichment in Bella 2021 E15.5 cortical query cells is robust to withheld reference cell types, reference downsampling, annotation granularity, and external reference atlases. A shows a horizontal barplot of G1 rates after systematically withholding each cortical cell type, with CThPN and Interneurons reaching 100% and Layer 4 and Ependymocytes showing low rates; B shows that ΔG1(Hom − Het) remains positive across five reference coverage levels from 30% to 100%; C shows grouped bars where Hom has higher G1 rates than Het at both fine and medium annotation granularities; D shows positive ΔG1(Hom − Het) across five independent cortical atlases, with the largest effect in Loo 2019 E14.5 and the smallest in Tsai 2024 E15.5.

**
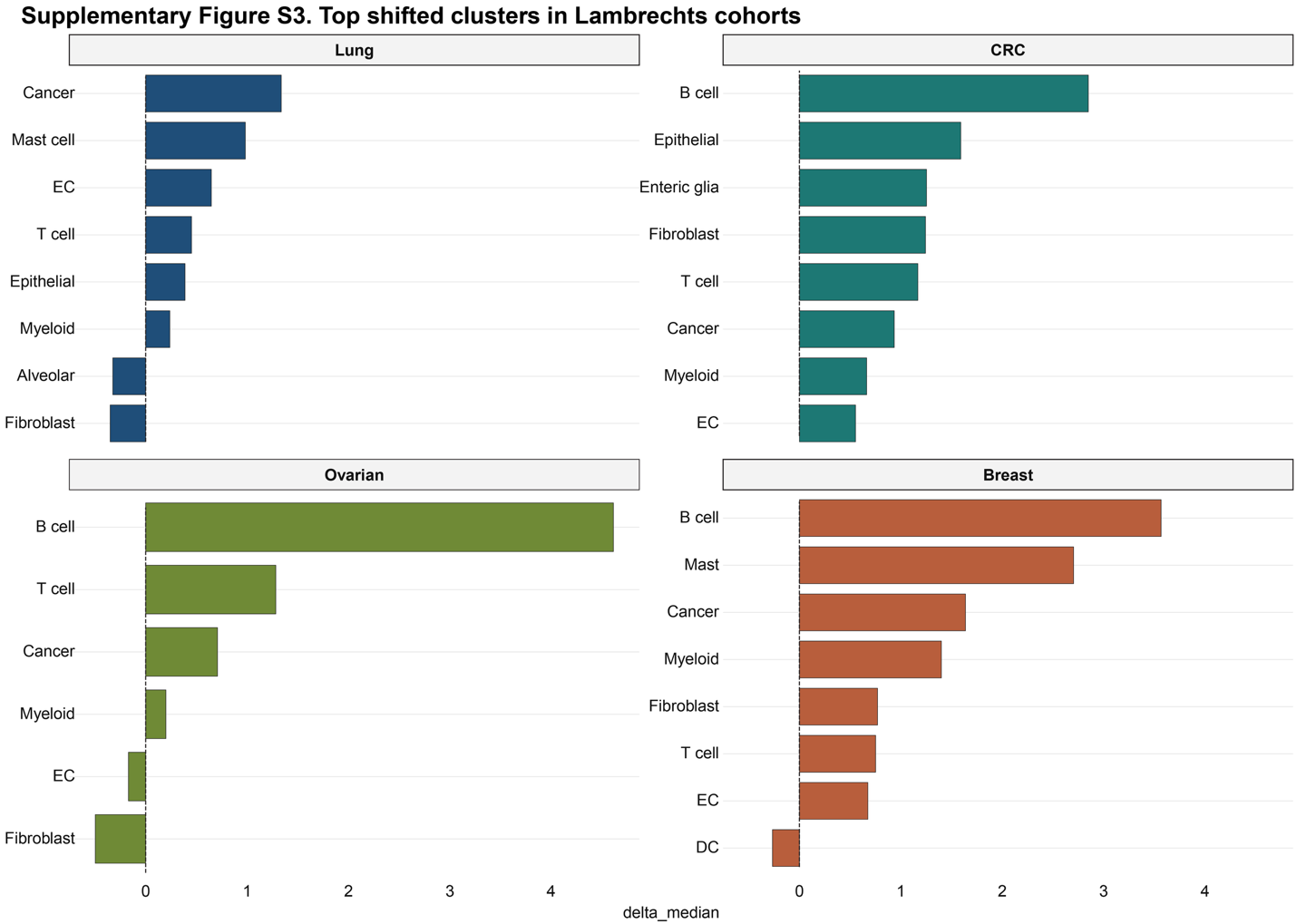
**

**Supplementary Figure S3.** Bar plots of top OOR-shifted clusters in the four Lambrechts cohorts (lung, colorectal, ovarian, and breast), ranked by delta median.

Alt text: Bar plots of top OOR-shifted clusters across lung, colorectal, ovarian, and breast cancer cohorts from the Lambrechts dataset, ranked by delta median values.


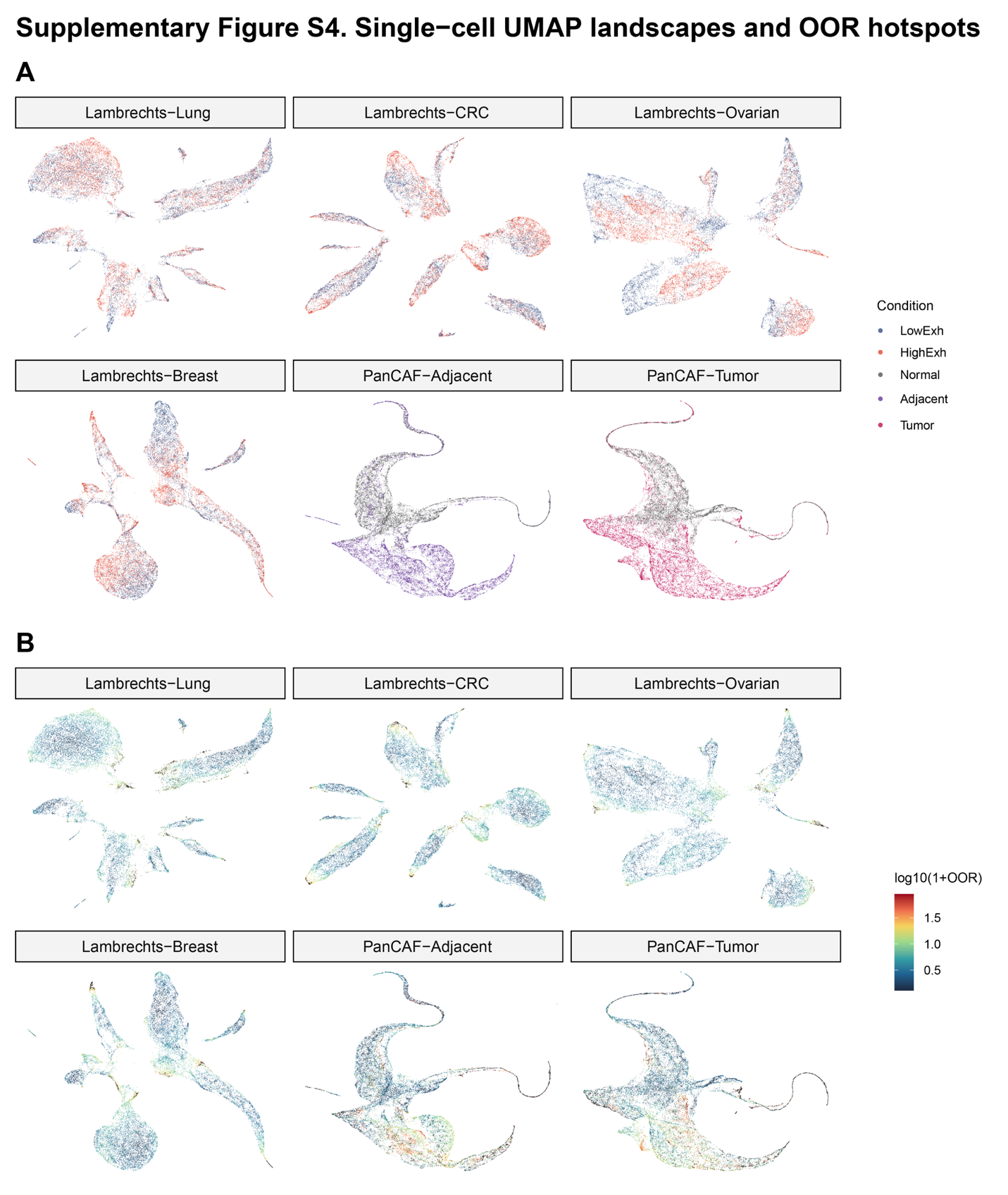


**Supplementary Figure S4.** Single-cell UMAP landscapes across all datasets, colored by comparison group and by OOR intensity, with high-OOR hotspots highlighted.

Alt text: UMAP landscapes of single-cell data across all analyzed datasets, colored by comparison group and OOR intensity, with high-OOR regions highlighted.


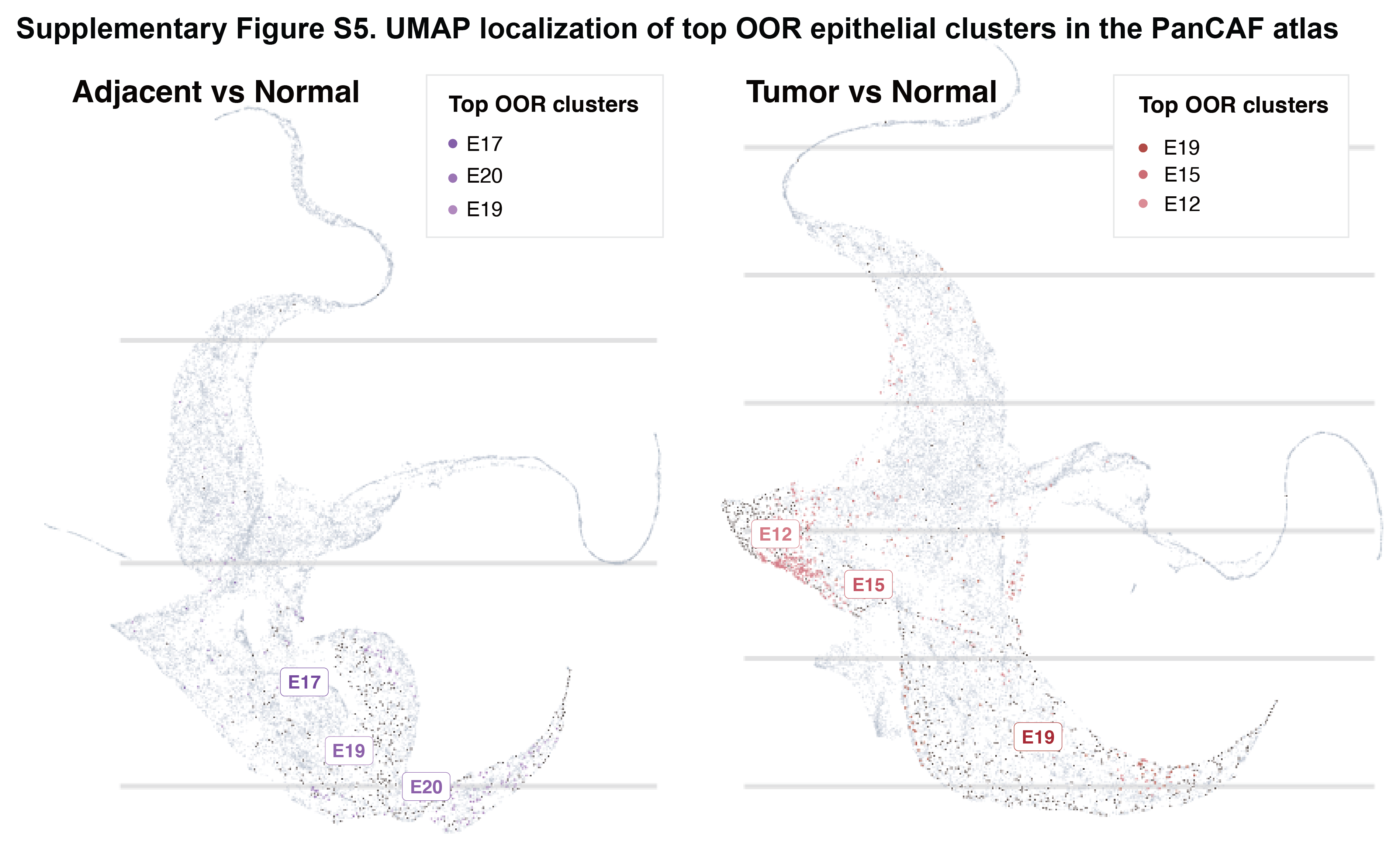


**Supplementary Figure S5.** Localization of top OOR epithelial clusters (Adjacent vs Normal, Tumor vs Normal) on the PanCAF UMAP. These clusters are concentrated within specific subregions, indicating focal instability hotspots.

Alt text: UMAP of the PanCAF epithelial atlas showing the spatial localization of top OOR clusters for Adjacent vs Normal and Tumor vs Normal comparisons, highlighting focal regions with elevated OOR signals.


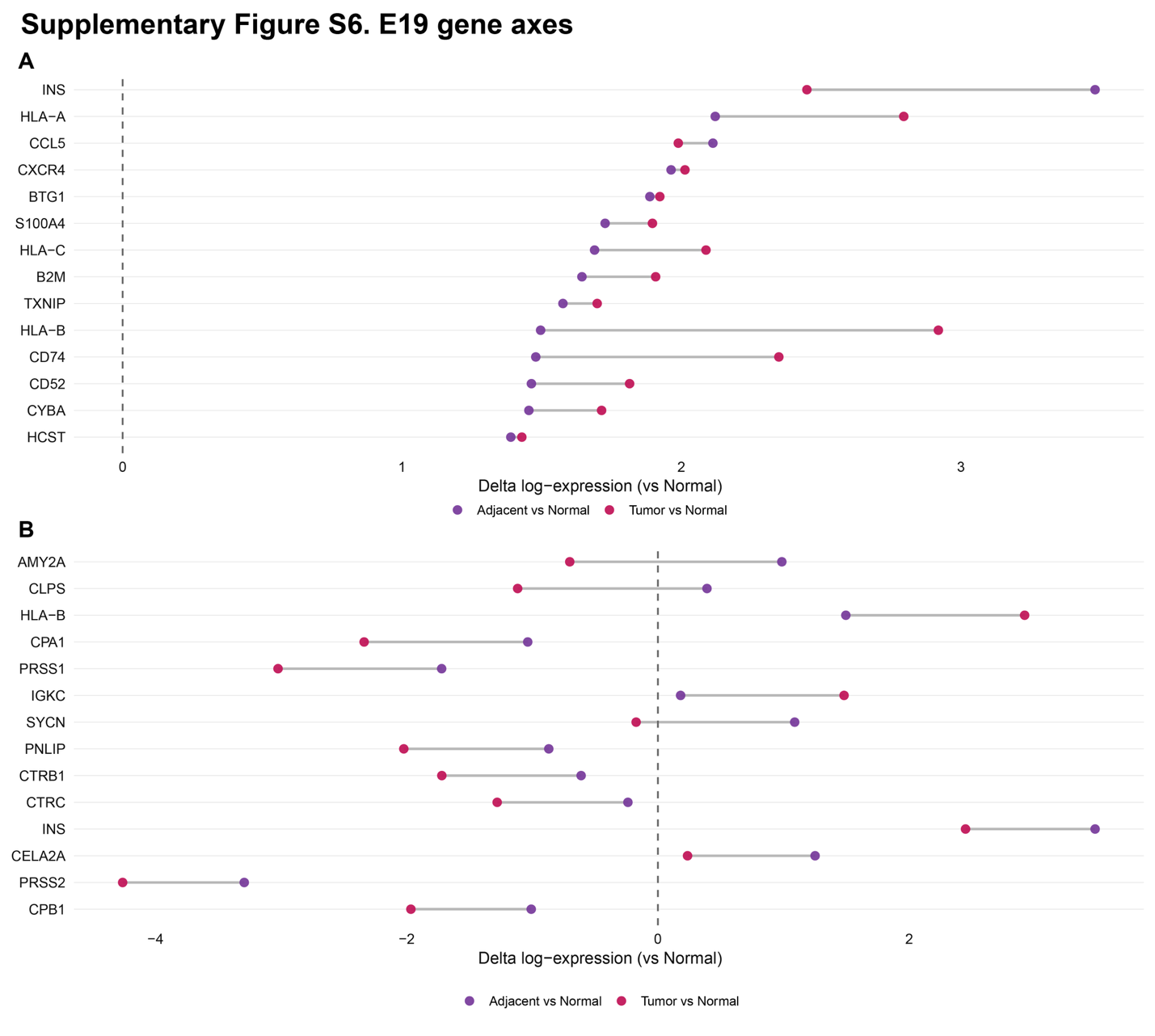


**Supplementary Figure S6.** E19 gene-level decomposition in PanCAF. **(A)** Genes showing concordant upshift in both Adjacent and Tumor versus Normal. **(B)** Genes showing maximal Adjacent–Tumor divergence relative to Normal.

Alt text: Two-panel gene-level decomposition of the E19 epithelial cluster in PanCAF. A shows genes with concordant upshift in both adjacent and tumor versus normal. B shows genes with maximal divergence between adjacent and tumor relative to normal.


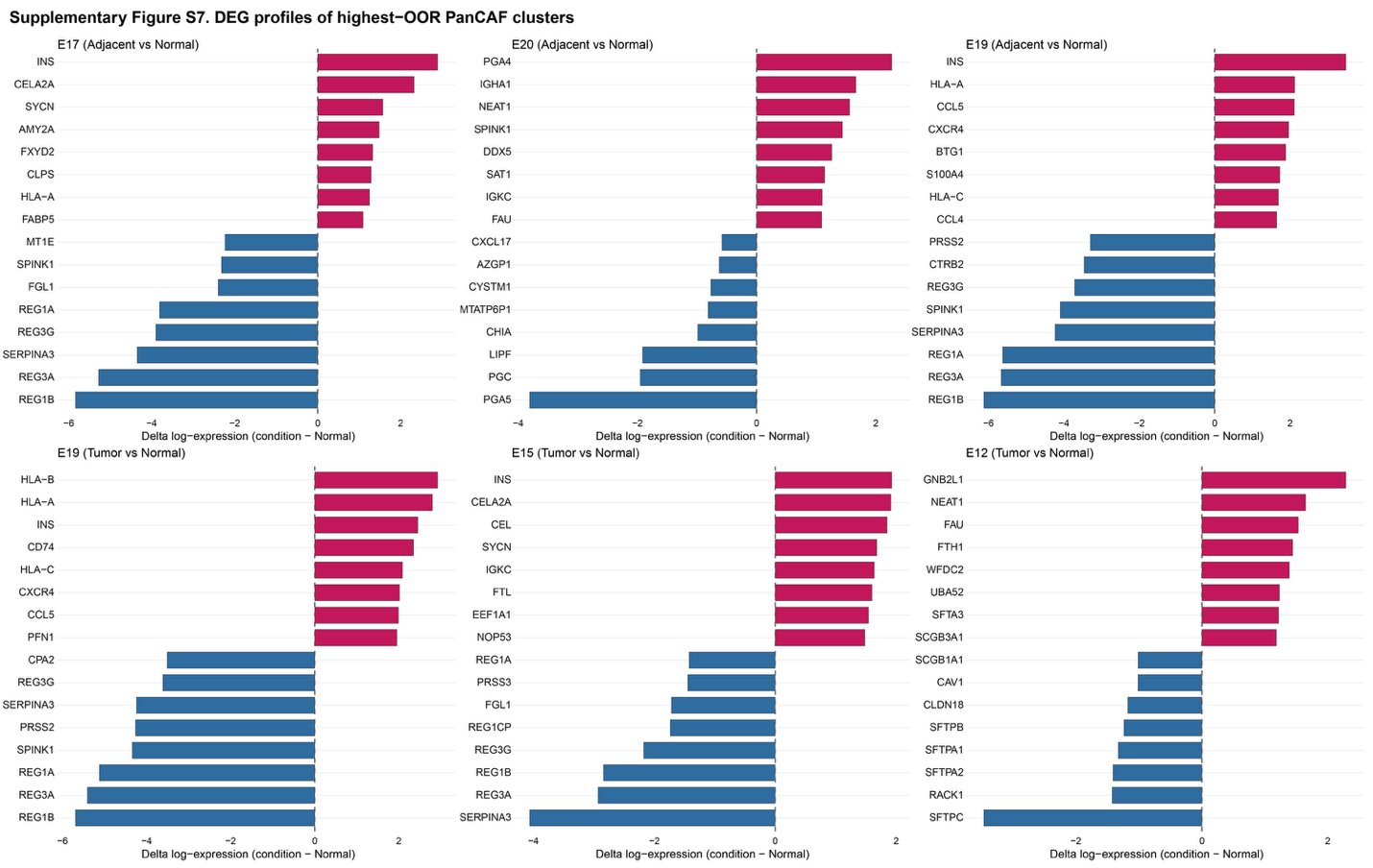


**Supplementary Figure S7.** DEG profiles of highest-OOR PanCAF clusters (E17/E20/E19 for Adjacent-vs-Normal and E19/E15/E12 for Tumor-vs-Normal), showing top up- and down-shifted genes per cluster.

Alt text: Differentially expressed gene profiles for the highest-OOR PanCAF epithelial clusters, showing top upregulated and downregulated genes per cluster in adjacent-versus-normal and tumor-versus-normal comparisons.
